# Using Neural Networks with Routine Health Records to Identify Suicide Risk: Feasibility Study

**DOI:** 10.2196/10144

**Published:** 2018-06-22

**Authors:** Marcos DelPozo-Banos, Ann John, Nicolai Petkov, Damon Mark Berridge, Kate Southern, Keith LLoyd, Caroline Jones, Sarah Spencer, Carlos Manuel Travieso

**Affiliations:** ^1^ Swansea University Swansea University Medical School Swansea United Kingdom; ^2^ Division of Intelligent Systems, Department of Computer Science Bernoulli Institute of Mathematics, Computer Science and Artificial Intelligence, Faculty of Science and Engineering University of Groningen Groningen Netherlands; ^3^ Cardiff Adult Self Injury Project Cardiff United Kingdom; ^4^ Hillary Rodham Clinton School of Law Swansea University Swansea United Kingdom; ^5^ Princess of Wales Hospital, Bridgend, ABMU Health Board Swansea United Kingdom; ^6^ Signals and Communications Department, IDeTIC University of Las Palmas de Gran Canaria Las Palmas de Gran Canaria Spain

**Keywords:** suicide prevention, risk assessment, electronic health records, routine data, machine learning, artificial neural networks

## Abstract

**Background:**

Each year, approximately 800,000 people die by suicide worldwide, accounting for 1–2 in every 100 deaths. It is always a tragic event with a huge impact on family, friends, the community and health professionals. Unfortunately, suicide prevention and the development of risk assessment tools have been hindered by the complexity of the underlying mechanisms and the dynamic nature of a person’s motivation and intent. Many of those who die by suicide had contact with health services in the preceding year but identifying those most at risk remains a challenge.

**Objective:**

To explore the feasibility of using artificial neural networks with routinely collected electronic health records to support the identification of those at high risk of suicide when in contact with health services.

**Methods:**

Using the Secure Anonymised Information Linkage Databank UK, we extracted the data of those who died by suicide between 2001 and 2015 and paired controls. Looking at primary (general practice) and secondary (hospital admissions) electronic health records, we built a binary feature vector coding the presence of risk factors at different times prior to death. Risk factors included: general practice contact and hospital admission; diagnosis of mental health issues; injury and poisoning; substance misuse; maltreatment; sleep disorders; and the prescription of opiates and psychotropics. Basic artificial neural networks were trained to differentiate between the suicide cases and paired controls. We interpreted the output score as the estimated suicide risk. System performance was assessed with 10x10-fold repeated cross-validation, and its behavior was studied by representing the distribution of estimated risk across the cases and controls, and the distribution of factors across estimated risks.

**Results:**

We extracted a total of 2604 suicide cases and 20 paired controls per case. Our best system attained a mean error rate of 26.78% (SD 1.46; 64.57% of sensitivity and 81.86% of specificity). While the distribution of controls was concentrated around estimated risks < 0.5, cases were almost uniformly distributed between 0 and 1. Prescription of psychotropics, depression and anxiety, and self-harm increased the estimated risk by ~0.4. At least 95% of those presenting these factors were identified as suicide cases.

**Conclusions:**

Despite the simplicity of the implemented system, the proposed methodology obtained an accuracy like other published methods based on specialized questionnaire generated data. Most of the errors came from the heterogeneity of patterns shown by suicide cases, some of which were identical to those of the paired controls. Prescription of psychotropics, depression and anxiety, and self-harm were strongly linked with higher estimated risk scores, followed by hospital admission and long-term drug and alcohol misuse. Other risk factors like sleep disorders and maltreatment had more complex effects.

## Introduction

### Background

The World Health Organization recognizes suicide as a public health priority. The World Health Organization Member States are committed to working towards the global reduction of suicide rates worldwide by 10% by 2020 [1]. In Wales alone, approximately 300 people die each year by suicide, accounting for about 1% of all deaths, and three times the rate of fatalities following traffic accidents [2]. The suicide rate has barely altered over the last decade, and any change that has occurred has generally been an increase [3]. Each death by suicide in the UK is estimated to cost more than £1,370,000 (direct and indirect costs) [4]. Considering these observations, adopting a public health approach to suicide prevention “has to be a national priority” [5].

Unfortunately, the prediction of suicide risk has proven to be a challenging problem for epidemiological studies and how they apply to health care practice. The pathways to suicide are mediated by highly complex processes, integrating many interdependent risk factor variables [6-9]. This creates difficulties around the positive identification of the relatively small number of individuals who will take their own lives from the much larger group of people in whom some or all the various risk factors have been identified. Assessment of immediate suicide risk requires a clinical evaluation. However, the majority of those who take their own lives present to health services other than mental health specialists in their final year. The identification of those at risk—so appropriate questions can be asked in relation to suicidality—would support ongoing suicide prevention efforts across a range of health services.

Short-term suicide risk prediction (ie, days, weeks, or months) is particularly useful for targeted interventions; but less is known about the processes underlying short-term suicidality than longer-term presentations [10]. Distal, or identified long-term risk factors, have complex effects on short-term risk and therefore separate, specific research is needed.

At the same time, we have databanks curating a wealth of electronic health records (EHRs), and administrative information which, when linked, could provide a representative picture of the biological, societal and health status of an individual at any point in time. Use of this data at scale is expected to make a pivotal contribution to the study of many diseases [11], especially those with complex longitudinal histories like suicide. However, the sheer volume of data and the complexity of the suicide factors-risk model have proven to be a challenge for traditional epidemiological and statistical modelling methods. As a result, existing screening tools are reportedly inefficient [12]. Thus, advanced artificial intelligence (AI) techniques are currently better positioned to tackle the combined challenges of big data and suicide risk prediction.

### Prior Work

Although the application of AI techniques in different areas of medicine is extensive [13,14], the difficulties of processing routinely collected EHRs and big data in general have been reported elsewhere [15-18]. These include the volume, complexity, heterogeneity and changing nature of medical data as well as its poor mathematical characterization; the importance of physician’s interpretations; and the legal, ethical and social implications. It is only recently that we have had the resources to record, maintain and analyze routinely collected EHRs with millions of records.

In the last decade, the use of machine learning (a branch of AI) to analyze EHRs has grown dramatically, spurred in part by advances in artificial neural networks (ANNs) and deep learning [19]. Miotto and colleagues [20] created a deep ANN that received hospital diagnosis codes and created a “patient representation” vector of 500 features. This vector was fed to a random forest to predict 78 different diseases, including mental disorders such as schizophrenia. This model generated an accuracy of more than 90% for (more than) 76,000 patients, but suicide risk was not part of the study.

Indeed, the application of AI in psychiatry is a field that has received relatively little attention but has great potential for innovation [11]. Some proposals found in the literature are optimization of the delivery of momentary cognitive-behavioral interventions [21], early identification of post-traumatic stress disorder [22], and analysis of social-network information for mental health research [23]. AI studies specifically focusing on suicide risk estimation are more recent and scarce.

Passos and colleagues [24] administered questionnaires to 144 participants with major depressive disorder or bipolar disorder to extract risk-factor information. Suicidality was estimated based on a previous history of suicide attempts. This data was then fed into various machine learning algorithms with the aim of identifying those at high risk of attempting suicide. A best performance of 72% accuracy was obtained with a relevance vector machine.

Kessler and colleagues [25] used a population cohort of non-deployed US Regular Army soldiers who had a diagnosed mental disorder and at least one outpatient visit. Their cohort included 147 deaths through suicide. Between 10 and 14 factors were extracted after outpatient visits followed by suicide (cases) and visits not followed by suicide (controls) and used to build a logistic regression with elastic net regularization to predict suicidality in the five weeks after these visits. Their system obtained a sensitivity of 48% and a specificity of 84% when predicting suicidality. The authors concluded that their system “outperformed mental health professionals to a large margin.”

### Goal of This Study

We aim to explore the use of ANNs with routinely collected EHRs to estimate suicide risk within the general population. This approach builds on Passos et al and Kessler et al research, taking it a step further by relying on routinely collected EHRs across health settings rather than mental health questionnaires. Hence, our system would not depend on information that is collected only in specific circumstances (eg, outpatient visits or hospital admissions), and could therefore be used to screen the entire population without increasing the workload of health care practitioners.

Our system aims to improve not only the quality of suicide risk assessment, but also its coverage. This is a crucial factor when considering that only 35% of those who died in Wales by suicide between 2010 and 2015, were admitted to hospital in the year prior to death, and around 40% had an emergency department admission. Furthermore, of those who died in Wales by suicide between 2001 and 2015, 65% did not have a mental health records in the year prior to death; and 40% never had. However, approximately 83% of these suicide cases had at least one contact with their general practitioner (GP) during that period. Therefore, our system seeks to utilize these contacts to assess suicide risk and increase population coverage.

Additionally, our system has the potential to perform risk assessment continuously over time and in the background (ie, without human intervention) across healthcare settings. Rather than using this as an assessment of immediate “at risk” or “not at risk,” it will be used to flag patients, even those attending for reasons other than mental health, so that appropriate questions can be asked. The UK National Institute for Health and Care Excellence recommends that risk assessment tools and scales should not be used to predict future suicide or repetition of self-harm [26]. This is because of the dynamic nature of suicide risk. An individual assessed as “not at risk” on one occasion could subsequently become “at risk,” but professionals may not be as responsive to these changes because of labelling effects. The proposed system aims to flag at risk individuals upon any contact with health services so that relevant questions are asked and appropriately addressed.

The goal of this study is to test the feasibility of this concept, validating the methodology from functionality (performance) and medical (validity of factors-risk model) points of view. Using an oversimplified system (shallow ANN), conservative results regarding model complexity and performance are ensured. We combine data from primary and secondary care, use repeated cross-validation during evaluation, and explore the distribution of factors across different levels of estimated suicide risk to describe the system’s behavior.

In the remainder of this article, we describe the data sources used, how we defined our cohorts of suicide cases and controls, and the risk factors used during experimentation. A brief introduction to ANNs is provided, followed by a detailed description of the models evaluated here. We detail the analyses that were run to assess raw performance and the resulting factors-risk model. Following the presentation of the results, we discuss their interpretation as well as the potential of the proposed model, how it compares with the current state of the art approaches, its limitations and implications for practice, and conclusions.

## Methods

### Materials

#### Data Sources

Data available within the Secure Anonymized Information Linkage (SAIL) Databank [27] was used. The HIRU Information Governance Review Panel (IGRP) granted ethical approval. IGRP is an independent body consisting of a range of government, regulatory and professional agencies, that oversee study approvals in line with permissions already granted to the analysis of data in the SAIL databank [28,29]. The current research took place under the SID-Cymru project [30] (approval number 0204).

For this study, we linked and analyzed the National Statistics Annual District Deaths Extract (ADDE), the Welsh Demographic Service (WDS), the Welsh Primary Care GP dataset (WGP), the Patient Episode Database for Wales (PEDW) and the Emergency Department Data Set (EDDS). While all datasets were used to define the study case-control cohort, only WDS, WGP and PEDW were used to build the feature vectors for experimentation.

Data availability varied across individuals and databases. While ADDE and PEDW datasets have a nationwide coverage, WPG contains data from 348 out of 474 (73%) GP practices in Wales. This variation was reduced by restrictions applied during the cohort definition (see below). At the same time, while the WGP and PEDW datasets were available over the full study period (2001 to 2015), ADDE was only available from 2009. However, ADDE data was used only to determine a key date before death, not to train or test the ANN system, and therefore we do not expect this has significantly biased our results.

#### Cohort Definition

We extracted our cohort from SID-Cymru, a population based electronic case-control study on completed suicide in Wales between 2001 and 2015 defined within SAIL [30]. Approximately 32,000 deaths of Welsh residents are registered each year, of which approximately 350 are suicides or events of undetermined intent. It is conventional research practice to include the latter in the definition of suicide [31].

The case-control study cohort was built according to the following steps:

1. We identified those that died through suicide at age 10 or older between 2001 and 2015. Deaths of undetermined intent in those under 10 years of age may be related to abuse or neglect and thus were excluded.
2. We followed individuals’ health histories retrospectively from death date to identify the full calendar of health services contact leading up to death (CLD). This could include multiple entries within the WGP, PEDW and EDDS databases (eg, attendance at A and E, admission to hospital, transfer to another hospital, and finally GP letters received from hospitals notifying of deaths). A maximum CLD duration of one month was considered to avoid including unrelated hospital stays. The CLD was subsequently removed from the analysis to avoid using information directly linked with the death of cases.
3. Only those residing in Wales at the time of their death, with GP data available for at least 80% of the five years prior to CLD were included in the study. This ensured that similar data coverage was available for all cases and controls. The value of five years was chosen to balance between the length of health history and number of cases retained.
4. For each case, 20 controls were randomly selected, without replacement and excluding cases, after matching by gender and week of birth (±1 year). During control selection, those with a similar period of Welsh residency and GP data coverage were prioritized to ensure similar coverage quality. Although this number is unnecessarily large for traditional paired case-control studies, the proposed methodology benefitted from increased data availability during training.

A total of 2604 suicide cases were identified—2012 (77.3%) of which were males, and 58,080 controls. These had a perfect (deterministic) or very high (probabilistic) linkage score (between 0.95 and 1) within SAIL.

#### Feature Vector

Only data from WDS, WGP and PEDW were used during experimentation. Not all events recorded in WGP and PEDW represent face-to-face contact with the patient, and a single event may have multiple associated entries (eg, multiple diagnoses).

Each entry was categorized in WGP and PEDW into types of health event: depression and anxiety; other common mental disorders; other mental health; non-intentional injury and poisoning; self-harm; alcohol misuse; drugs misuse; possible maltreatment; physical sleep disorders; non-physical sleep disorders; and “others.” We also identified the prescription of opiates and psychotropics from WGP (PEDW has no prescription information) and recorded whether there were any entries recorded in WGP or PEDW (representing a hospital admission). This made a total of 15 factors (11 diagnoses, two prescriptions, WGP entries and hospital admissions).

The above categories were defined in terms of ReadCodes for WGP and ICD10 for PEDW with the help of expert clinicians and based on previous publications when available (depression and anxiety [32], other common mental disorders [33], non-intentional and intentional (self-harm) injury and poisoning [34,35], alcohol misuse [36], drugs misuse [36,37], possible maltreatment [38] and psychotropics [39]). Full code definitions can be seen in Tables A1 and A2, Multimedia Appendix 1.

We identified the presence of the above 15 health events during four non-overlapping time-frames:

- 1M: Between CLD and 1 month before CLD [CLD – 1 month, CLD].
- 6M: Between 1 and 6 months before CLD [CLD – 6 months, CLD – 1 month).
- 1Y: Between 6 and 12 months before CLD [CLD – 1 year, CLD – 6 months).
- 5Y: Between 1 and 5 years before CLD [CLD – 5 years, CLD – 1 year).

The final feature vector also included age at CLD and sex, resulting in a length of 62: 1 float age + 1 binary sex + 15 binary health events * 4 time-frames. This feature vector does not include data directly related to the CLD. Interactions between these factors are automatically taken into account by the ANN.

### System Design

#### Artificial Neural Networks

Artificial neural networks (ANNs) are biologically inspired computing systems capable of learning tasks through examples and experience, without the need of explicit programming of task-specific rules or any a priori knowledge of the solution [40].

**Figure 1:**
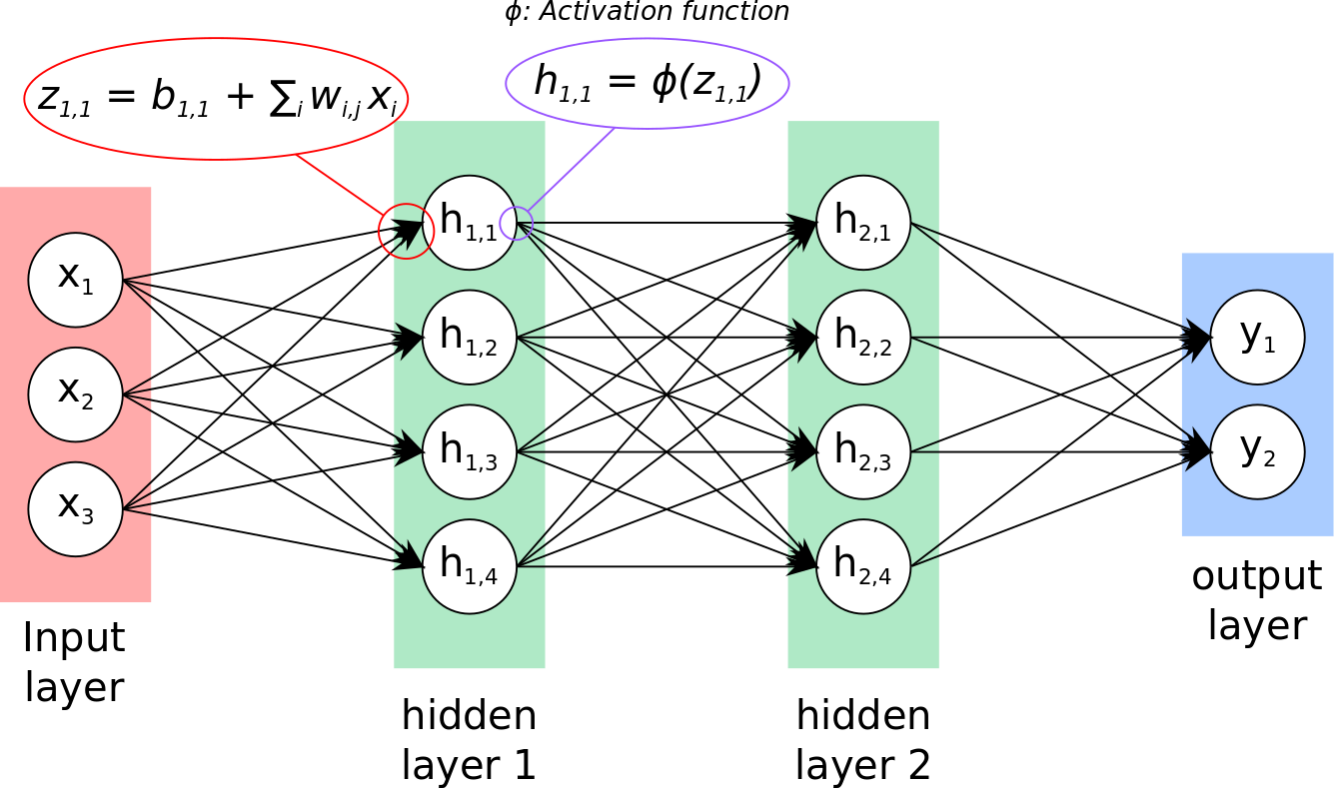
Structure of an artificial neural network with 1 input layer (red), 2 hidden layers (green) and one output layer (blue) all fully connected.

ANNs are typically composed of an input layer, one or more hidden layers and an output layer (Figure 1). Each unit (artificial neuron) in the input or output layer corresponds to one dimension of the input or output vector respectively, with each dimension corresponding to one input or output variable. The complexity of the input-output model is governed by the activation function of neurons, the number of hidden layers, the number of neurons in each layer and the connection between neurons and layers.

The term “black-box” is sometimes used to describe ANNs. This has contributed to the widespread misconception of ANNs not being transparent, which in turn has gained them a bad reputation in fields such as medicine, where understanding how and why decisions are taken is important. However, “black-box” alludes to the fact that the input-output model generated by the network is too complex to be expressed by a set of simple rules that are syntactically meaningful. Such a model can nevertheless be expressed as a mathematical equation. For example, a simple ANN composed of no hidden layers and a single output neuron with a logistic activation function is equivalent to the logistic regression model;


*y* =
*S*(
*b* + ∑
_∀
*i*_
*w
_ji_ x
_i_*)

where *x*_i_ are each of the input neurons (ie, variables), *w*_ji_ are the weights from the *i*-th input to the *j*-th neuron, *b* is a bias term, *S(∙)* is the sigmoid function and *y* is the output neuron (ie, result). Typically, the input-output equation quickly grows in complexity, and therefore we opt not to represent it.

#### Evaluated Architecture

A simple ANN was implemented with seven different configurations: no hidden layers (nn0), one hidden layer of size 10, 50 or 100 (nn10, nn50, nn100) and two hidden layers with sizes 10, 50 or 100 (nn10-10, nn50-50, nn100-100). All layers were fully connected (ie, each neuron in layer *i* was connected to all neurons of the previous layer *i-1*). The input layer was composed of the feature vector described above (ie, 62 neurons). Hidden layers, when present, had a tanh activation function. The output layer had a single neuron with a sigmoid activation function, returning the score *r* of a sample belonging to a (suicide) case (*r=1*) or a control (*r=0*). A decision threshold of 0.5 was used, ie, samples with *r>0.5* were classified as cases while samples with *r* ≤*0.5* were classified as controls. We interpreted this score *r* as the estimated risk of suicide, differentiating between very low risk (VLR; *r* ≤0.17), low risk (LR; *0.17<r* ≤*0.33*), moderate-low risk (MLR; *0.33<r* ≤*0.5*), moderate-high risk (MHR; *0.5<r* ≤*0.67*), high risk (HR; *0.67<r* ≤*0.83*) and very high risk (VHR; *r>0.83*).

The mean square error was adjusted to account for data imbalance (20 controls per case) so that the resulting cost of both classes (case and control) was equal to 1. The final cost included l2 weight regularization with scale 0.01.

All ANNs were trained with the gradient descent algorithm and exponential learning rate decay starting at 1. Training was performed sequentially with three different batch sizes: 25, 100 and all cases and their respective controls (ie, total batch size 525, 2100 and full). The learning rate was reset with every change in batch size. Training within each batch size continued until a maximum number of epochs was reached, the change of cost function evaluated on the validation set was lower than a threshold or the change was in the negative direction (ie, not improving).

Using the oversimplified system (ie, small number of features and shallow ANNs) described above, we favored obtaining conservative results in terms of model complexity and performance, which we hope would counteract some of the limitations of the study (described below). In addition, in a practical application the cost of misidentifying suicide cases and controls will probably not be the same. Whether the system should be tuned to have a high sensitivity at the cost of low specificity or vice versa depends on many factors and is beyond the scope of this study. For simplicity, we equalized this cost to be the same for cases and controls. Hence, accounting for the unbalanced 1:20 distribution of cases and controls, the cost of misclassifying a case was 1, while the cost of misclassifying a control was 1/20. All experiments and ANNs were designed and executed using TensorFlow in Python.

### Statistical Analysis

#### System Performance

We followed a 10x10-fold cross-validation approach to evaluate the performance of the ANNs. On each iteration, one-fold was used for testing, one for validation (used to inform the early stopping training algorithm) and eight for training. Cases were randomly distributed across folds, followed by their respective controls so that case-control pairs were always maintained during partitioning (this partitioning rule was also applied during batch partitioning in training).

On each iteration, as well as measuring the classification error obtained with the threshold resulting from training, the threshold was varied to compute the receiving operating characteristics (ROC) curve and the area under the ROC curve (AUC). We compared performance between systems using a corrected resampled *t* test [41] based on the average over sorted runs [42] for 10x10-fold, and *P* values were further adjusted (*Q* values) for multiple testing using the false discovery rate Benjamini and Hochberg method [43].

Finally, we repeated the above analysis shuffling the labels of each samples, ie, we randomly assigned the label “case” to one of the 20 paired controls of a case and rebranded the original case as “control,” This aims at evaluating whether our initial results are due to real relationships between labels and data, rather than to random idiosyncratic patterns in the data.

#### System Behavior

In addition to measuring system performance, we attempted to assess the factors-risk model obtained by the best performing ANN. Due to the dimensionality of the feature vector (ie, number of input factors) and the freedom of the ANN to build complex models with numerous non-linear interactions, getting the full representation of the factors-risk model was not practical. However, the following results gave us insight into how large a role each factor played in the computation of the risk score:

- The histogram of the number of cases and controls across estimated risk scores. This will provide information additional to the performance measurements about the classification capability for cases and controls.
- The histogram of the estimated risk difference when turning specific factors “on” and “off’” across the whole dataset. This will show an estimated role of each individual factor in the computation of the risk score, and how it varies due to interactions with other factors.
- The distribution of each factor (ie, individuals presenting a factor) across estimated risk scores. This will work in conjunction with the previous point to draw an estimate of the role of each individual factor.
- The incidence of each factor within estimated risk scores. This will allow us to compare incidences across risk levels for cases and controls.

Results of this analysis refer to the factor-risk model built by our ANN and do not necessarily agree with the real factor-risk model. Our confidence of how similar these two are depends on the size and quality of the testing data and on the performance of our system. This is true for any AI application, but it is especially important in medical applications such as the one proposed here.

## Results

### System Performance

The error rate of the described ANNs decreased slightly from 28.9% to 26.8% when increasing the number of hidden layers from 0 to 2 (Table 1). Overall, nn0 performed worse than the rest. The performance difference between networks with 1 and 2 hidden layers, although small, is statistically significant (*q<* 0.05) (Table A3 of Multimedia Appendix 1).

**Figure 2:**
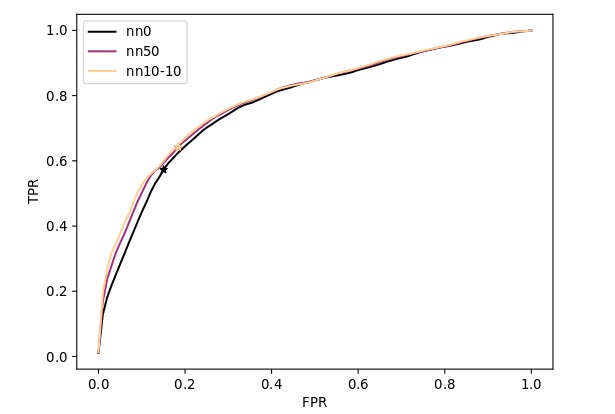
Receiving operating characteristics (ROC) curve for nn0, nn50 and nn10-10. FPR: false positive rate; TPR: true positive rate; nn0: no hidden layers; nn50: 1 hidden layer with 50 neurons; nn10-10: 2 hidden layers with 10 neurons.

Figure 2 shows the ROC curve of the best performing network for each number of hidden layers (ie, nn0, nn50 and nn10-10). ROC curves of nn10, nn50 and nn100 were virtually identical, as were curves of nn10-10, nn50-50 and nn100-100. In the false positive rate (fpr=1-specificity) range between 0 and 15%, nn50 and nn10-10 perform better than nn0. Past this point, the ROC curves get closer together and for fpr>30% they become virtually identical. Despite the similarity between ROCs of nn50 and nn10-10, the difference in AUCs between them is statistically significant (*q<* 0.05) (Table A4 of Multimedia Appendix 1). In general terms, nn10-10 and nn50 can obtain better sensitivity for more restrictive specificity values than nn0 but perform similarly well for higher specificity.

Crucially, results after shuffling the labels were characteristic of a random process (ie, 50% error rate and 0.5 AUC).

### System Behavior

**Figure 3:**
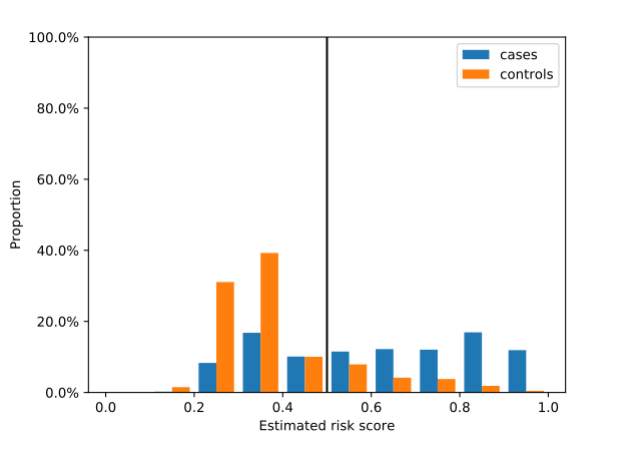
Distribution of cases and controls across estimated risk score levels. Those with risk score >0.5 were identified as “cases.”

The distribution of cases and controls across estimated risk scores reflects the results of Table 1 (Figure 3). Controls were mostly concentrated on scores below 0.5 (hence, high specificity). Cases on the other hand were almost uniformly distributed (hence, low sensitivity). Overall, few individuals received an estimated risk score ≤0.2.

**Figure 4:**
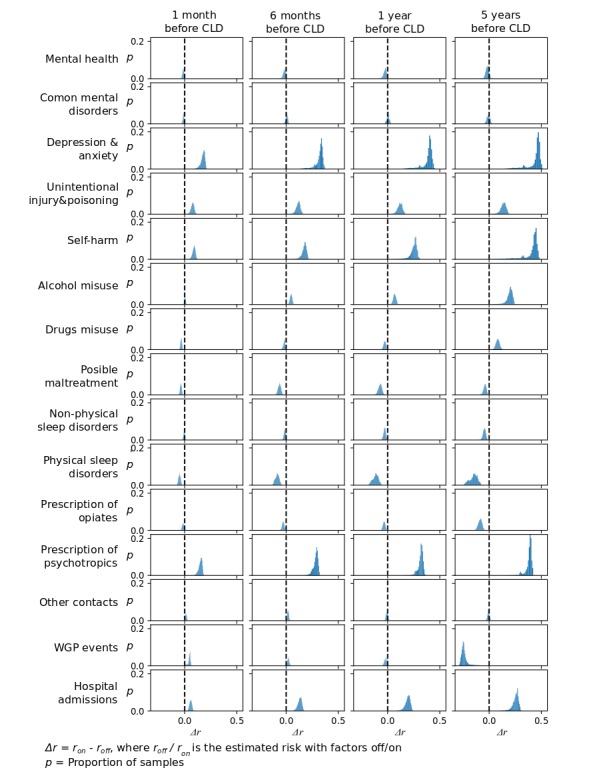
Histogram of the difference in estimated risk score when turning specific factors ‘on’ and ‘off’ across the whole dataset. CLD: contact leading up to death.

Prescription of psychotropics, depression and anxiety, and self-harm seem to have the strongest effect on the estimated risk, increasing *r* by ~0.4 when changing from “off’” to “on” across all time-frames (Figure 4). Most of the risk increase from prescription of psychotropics and depression and anxiety came on the first six months before CLD (∆*r≈* 0.3), while self-harm had a more linear effect across time-frames. The distribution of ∆*r* for prescription of psychotropics was the most concentrated around the peak. These three factors were followed in strength by hospital admissions and alcohol misuse, with ∆*r≈* 0.25. WGP entries, on the other hand, reduced the estimated risk by around 0.2.

Most samples were assigned a risk below the 0.5 threshold, with only 70 individuals resulting in a a very low risk r≤0.17 (Table 2). In contrast, as many as 1366 individuals obtained a very high estimated risk (*r*>0.83). Age and gender distributions were virtually identical across risk levels, except for the very low risk range (*r* ≤0.17) which was mainly composed of women (Table 2).

**Figure 5:**
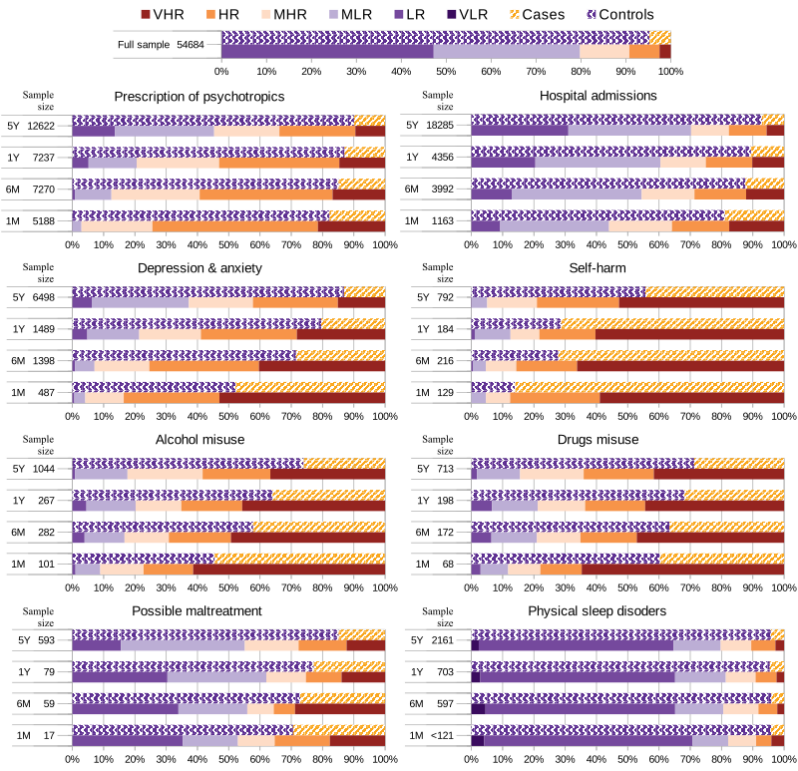
Samples presenting a specific factor and their distribution across cases and controls, and across estimated risks from very low (VLR) to very high (VHR). To the left of each bar group, the total number of individuals presenting the factor (sample size). At the top, the distribution of the full population. VHR: very high risk (r>0.83); HR: high risk (0.67<*r* ≤0.83); MHR: moderate-high risk (0.5<*r* ≤0.67); MLR: moderate-low risk (0.33<*r* ≤0.5); LR: low risk (0.17<*r* ≤0.33); VLR: very low risk (*r* ≤0.17).

Looking at how factors (individuals with factors “on”) were distributed across risk scores (Figure 5, and Tables A5 to A8 of Multimedia Appendix 1), in the month before CLD, 97% of those with a prescription of psychotropics, 96% of those with depression and anxiety and 95% of those with self-harm were classified as being at risk of suicide (*r*>0.5) (Figure 5). More than 78% of those presenting with one of these factors or drugs or alcohol misuse across most of the considered time-frames (i.e. 1M, 6M, 1Y and 5Y) were classified as at risk. Moreover, more than half of the individuals with recorded self-harm in the five years before CLD, or depression and anxiety or alcohol or drugs misuse in the month before CLD, received a very high estimated suicide risk score (r>0.83).

**Table 1:** Mean and standard deviation of the error rate, sensitivity, specificity and AUC obtained on the 10x10-fold experiments for each neural network.

ANN^a^ model	Error rate, mean (SD)	Sensitivity, mean (SD)	Specificity, mean (SD)	AUC^b^, mean (SD)
nn0^c^	28.89% (1.47)	57.28% (2.97)	84.94% (0.54)	0.78 (0.02)
nn10^d^	27.12% (1.42)	64.19% (2.94)	81.57% (0.57)	0.79 (0.02)
nn50^e^	27.09% (1.42)	64.25% (2.92)	81.57% (0.58)	0.79 (0.02)
nn100^f^	27.11% (1.42)	64.18% (2.93)	81.61% (0.61)	0.79 (0.02)
nn10-10^g^	26.78% (1.46)	64.57% (3.00)	81.86% (0.58)	0.80 (0.02)
nn50-50^h^	26.83% (1.43)	64.52% (2.92)	81.82% (0.59)	0.80 (0.02)
nn100-100^i^	26.83% (1.47)	64.54% (3.04)	81.79% (0.61)	0.80 (0.02)

^a^ANN: artificial neural network.

^b^AUC: area under the ROC curve.

^c^nn0: No hidden layers.

^d^nn10: 1 hidden layer with 10 neurons.

^e^nn50: 1 hidden layer with 50 neurons.

^f^nn100: 1 hidden layer with 100 neurons.

^g^nn10-10: 2 hidden layers with 10 neurons.

^h^nn50-50: 2 hidden layers with 50 neurons.

^i^nn100-100: 2 hidden layers with 100 neurons.

**Table 2:** Number of individuals, gender and mean age for controls, cases and estimated risk levels from very low to very high.

Description	Number of Individuals	Number of Males, n (%; 95% CI)	Mean age, years
Controls	52080	40240 (77.37%; 76.9%-77.6%)	48.04
Cases	2604	2012 (77.27%; 75.9%-78.6%)	48.04
Very low risk (*r* ≤0.17)	70	4 (5.7%; 2.6%-12.1%)	54.32
Low risk (0.17<*r* ≤0.33)	25744	17884 (69.5%; 68.9%-69.9%)	48.07
Moderate-low risk (0.33<*r* ≤0.5)	17818	15850 (88.9%; 88.6%-89.3%)	46.52
Moderate-high risk (0.5<*r* ≤0.67)	6011	4765 (79.3%; 78.4%-80.1%)	49.31
High risk (0.67<*r* ≤0.83)	3675	2703 (73.5%; 72.3%-74.7%)	53.03
Very high risk (*r*>0.83)	1366	1046 (76.6%; 74.6%-78.4%)	47.75

**Figure 6:**
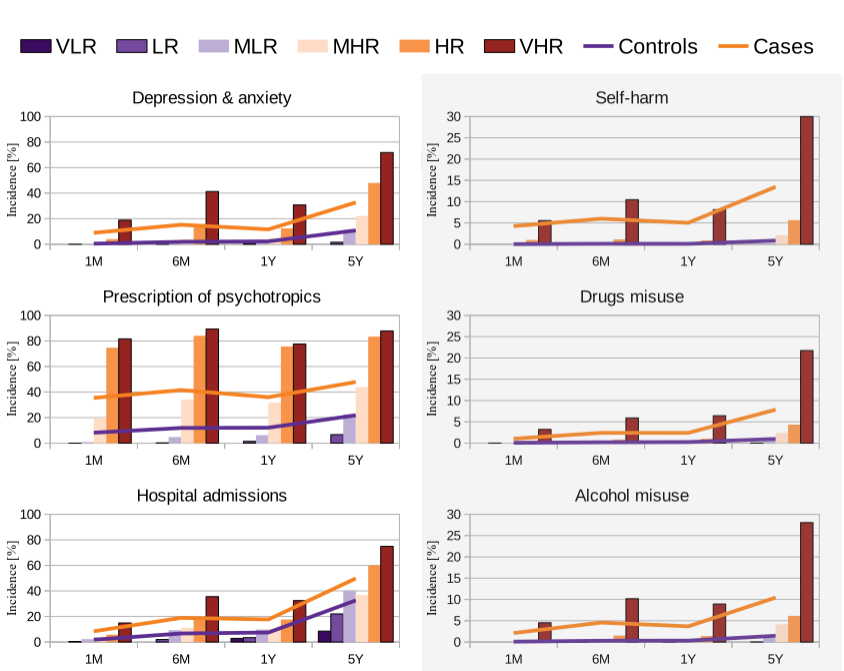
Incidence of factors for cases, controls and estimated risk levels from very low (VLR) to very high (VHR). Panels on the right hand column (shaded) have y-axis limits between 0% and 30% to facilitate visualization. VHR: very high risk (r>0.83); HR: high risk (0.67<*r* ≤0.83); MHR: moderate-high risk (0.5<*r* ≤0.67); MLR: moderate-low risk (0.33<*r* ≤0.5); LR: low risk (0.17<*r* ≤0.33); VLR: very low risk (*r* ≤0.17).

In terms of incidence (Figure 6, and Tables A9 to A12 of Multimedia Appendix 1), prescription of psychotropics across time-frames had an incidence between 77% and 90% on those with very high risk (r>0.83), and lower than 7% on those not at risk (*r* ≤0.5), except on the 5Y period, which had an incidence of 22% on those with moderate-low risk (*0.33<r* ≤*0.5*) (Figure 6). In comparison, between 35% and 48% of actual cases presented this factor. More than 70% had a depression and anxiety event and a hospital event between one year and five years before CLD.

## Discussion

### Principal Results

The presented oversimplified system successfully differentiated between 2604 suicide cases and 52,080 matched controls in 73.22% of tested instances during 10x10-fold cross-validation. It achieved this using only routinely collected EHRs from GP and hospital admissions in the five years before the case’s CLD.

The reduction in error rate as the number of hidden layers increased is representative of the complexity of the underlying suicide factors-risk model. In our case, results barely changed when the number of neurons in the hidden layers increased. In fact, performance differences between networks with the same number of layers came from a better tuning of the output scores resulting in an operational point closer to the optimal (ie, equal error rate). Overall, we expect the advantages of having more layers and neurons to become obvious when more factors are fed into the model.

The disparity that was observed between sensitivity and specificity and on the score distribution between cases and controls highlights the variation in the level of difficulty experienced when analyzing both groups. Controls seem to follow more uniform patterns and are therefore easier to identify, hence the higher specificity and the clustering of controls below a 0.5 score. On the other hand, patterns of the cases are more heterogeneous, with some having feature vectors identical to controls, which explains the lower sensitivity and the almost uniform distribution of cases across risk scores.

The presented behavioral evaluations do not unequivocally explain the factor-risk model built by the network. However, they do provide a general idea of what is driving the output score upwards. The input factors prescription of psychotropics, depression and anxiety, and self-harm, and, to a lower degree, drugs and alcohol misuse, were strongly linked with increasing estimated risk scores. This is in keeping with previous literature [6-8] and provides evidence for proof of concept and the feasibility of identifying high risk individuals using ANNs and routinely collected EHRs. Similarly, gender and age were not related with risk estimation, also in line with findings of short-term risk studies [10].

On the other hand, some risk factors identified in the literature did not exhibit the same behavior in our results. Physical sleep disorders seemed to decrease rather than increase the estimated risk. Due to the relatively low incidence of this factor in our data, its effect may be attenuated by and highly dependent on more active factors. This would also explain the dispersion of its effect on the estimated risk score (Figure 4). Furthermore, possible maltreatment also seemed to reduce the estimated risk. However, after a closer look, its effect seems to change sign as the maltreatment gets further away from the CLD, with possible maltreatment in the 5Y time-frame increasing the estimated risk. This may be related to long lasting effects of maltreatment and with help and support received in the first year after the maltreatment.

Due to the non-perfect specificity and relatively low sensitivity obtained, results from the behavioral analysis should not be directly extrapolated to the real-world factor-risk model. Having said that, the remarkable agreement between our model and the existing literature works as an indication of the feasibility of our proposal. Additionally, we expect to substantially improve performance with a more complex system design, which will in turn increase our confidence in the validity of the obtained factors-risk model.

### Potential of the Proposal

Perfect estimation of suicide risk using EHRs will never be possible, mainly because some individuals take their own life without ever seeking help or without presenting to health care services with signs of being at risk. In addition, of those that seek help or present with evidence, signs may be missed or inaccurately or insufficiently recorded. Others may simply present insufficient evidence to distinguish them from controls (ie, having a pattern identical to controls).

According to our data, around 90% of those that died through suicide in Wales had one or more contacts with health services in the year prior to their CLD, and approximately 30% of them had a contact related to their mental health. Therefore, the proposed methodology still has a good scope for application.

### Comparison with Prior Work

To our knowledge, Passos’ [24] and Kessler’s [25] are the only two publications to date with proposals comparable to ours. They reported 72% and 66% of accuracy respectively, compared to 73% obtained by our best system. However, these results cannot be directly compared due to differences in the application setting, data used and the evaluation process. Firstly, they applied and tested their systems in a hospital setting with only mental health patients. Secondly, their systems used smaller datasets and data extracted from questionnaires or outpatients visits with a specialist. Here we used diagnoses in primary and secondary care which are less specific, and primary care records have little indication of severity.

Interestingly, while Kessler’s method also suffered from low sensitivity, Passos’ system obtained comparable sensitivity and specificity. This may be due to the latter using data from the questionnaire Structured Clinical Interview for DSM-IV axis-I Disorders, which records highly specific diagnoses. In addition, Passos’ system aimed at differentiating previous suicide attempters from non-attempters, rather than identifying future risk.

### Limitations

The results presented here are limited by the purposely oversimplified system design used both in terms of the number of factors considered (only 15 over four time-frames) and the design of the ANN (a maximum of two hidden layers). Still, our system improved chance identification by almost 50%. As we move from feasibility to pilot study and increase the complexity of the system we expect to increase performance substantially.

The problem of suicide risk estimation suffers not only from a highly complex factors-risk model, but also from a lack of a quantitative measure of the real risk of suicide which is only known with certainty within a short time span before a recorded attempt. At any other time-point, we do not know the real risk for any individual. Someone at risk may refrain from ever attempting suicide, whereas another person may become at risk and attempt suicide within a very short period. This will have implications for a more practical evaluation (compared to the feasibility analysis presented here), as we will need to find ways to assess performance fairly without knowing the real risk ourselves.

Without properly labelled data, we need to rely on clinicians to assess the factors-risk model constructed by the algorithm. In our case, most of the individuals with a self-harm event were classified as cases or as being at risk (ie, r>0.5). Some of them belonged to the control group, and we considered these as errors in our evaluation. However, should all these instances be considered errors? The answer to this question is not trivial, and has technical, clinical and ethical implications that we need to explore in more depth.

### Implications for Practice

Our proposal will be most practical in settings where professionals do not have specialist mental health training but are in contact with individuals at risk of suicide. Nurses, emergency department staff, ambulance services, police and prison workers would be among the ones benefiting the most from the tool proposed here. These professionals face both the challenge of seeing large numbers of people where it is difficult to discern those at risk, and of assessing the suicidality of individuals often without having received sufficient training and under staff shortages [44,45]. As a result, it can be a challenge to identify individuals for appropriate assessment and care [46]. Having an advanced assessment tool with complex factors-risk models that produces good estimations would be invaluable in these cases.

### Conclusions

Prescription of psychotropics, depression and anxiety, and self-harm were strongly linked with higher estimated risk scores, followed by hospital admissions and long-term drugs and alcohol misuse which is in keeping with the current literature. Other risk factors such as sleep disorders and maltreatment had more complex effects.

The system presented here is an oversimplified one, using a short feature vector and shallow ANNs to assess the practicality of using EHRs in this way. As a feasibility study, we are more interested in (a) confirming the existence of discriminant information, and (b) validating the proposed methodology, than obtaining high accuracy rates. Nevertheless, our system obtained an accuracy like other published methods based on specialized questionnaire data.

Prescription of psychotropics, depression and anxiety, and self-harm were strongly linked with higher estimated risk scores, followed by hospital admissions and long-term drugs and alcohol misuse. Age and gender had no effect on risk. Interestingly, possible maltreatment had the opposite effects in the short and long terms, decreasing risk when recent and increasing it when more than a year before CLD.

The promising performance obtained with a basic ANN, and the fact that the resulting factors-risk model was in line for the most part with the literature, supports the hypothesis of the possibility of building a tool capable of estimating suicide risk in the general population using only routinely collected EHRs. We are a long way from employing such methods in clinical practice, but this is a first step to harness the potential of routinely collected electronic health records to support clinical practice in real time.

## Appendix

Multimedia Appendix 1 Supplementary tables.

## Abbreviations

ADDE: National Statistics Annual District Deaths Extract
AI: artificial intelligence
ANN: artificial neural networks
AUC: area under the ROC curve
CLD: contact leading to death
EDDS: Emergency Department Data Set
EHR: electronic health records
GP: general practice
HR: high risk (0.67<r≤0.83)
LR: low risk (0.17<r≤0.33)
MHR: moderate-high risk (0.5<r≤0.67)
MLR: moderate-low risk (0.33<r≤0.5)
PEDW: Patient Episode Database for Wales
ROC: receiving operating characteristics
SAIL: Secure Anonymised Information Linkage databank
VHR: very high risk (r>0.83)
VLR: very low risk (r≤0.17)
WDS: Welsh Demographic Service
WGP: Welsh Primary Care GP dataset
