# Supplementary material for: Using Neural Networks with Routine Health Records to Identify Suicide Risk: Feasibility Study
Source: JMIR Ment Health. 2018 Jun 22;5(2):e10144. doi: 10.2196/10144 (PMC6035342; doi:10.2196/10144)
Supplement: Multimedia Appendix 1 [file mental_v5i2e10144_app1.pdf]

Using neural networks with routine health records to identify suicide risk

Multimedia Appendix 1

Table A1: ICD10 definitions used.

	ICD10
Alcohol misuse	F10, K70, E244, G312, G621, G721, I426, K292, K852, K860, Y573, Z502, Z714, Z721
Common mental disorder	F30-F48
Depression and anxiety	F32, F33, F251, F341, F412, F40, F41
Drugs misuse	F1, R782, R783, Z503, Z715, Z722<br>Except: F10, F17
Injury and poisoning	X60-X84, Y10-Y34, Y870, Y872, Z915, V01-X59, X85-Y09, W75, W76, X40-X49<br>Except: W75, W76, X40-X49
Mental health	F
Self-harm	X60-X84, Y10-Y34, Y870, Y872, Z915

Table A2: ReadCode definitions used.

	ReadCodes
Alcohol misuse	66e, 9k1, E01, E23, du1, du5, 136K, 136S, 136T, 136W, 13Y8, 1462, 1B1c, 38Dz, 8BA8, 8G32, 8H35, 8H7p, 8HHe, 8HkG, 8HkJ, 8IAF, 8IAJ, 8IAt, 8IEA, 9NN2, E250, Eu10, F25B, F375, G555, J153, J610, J611, J612, J613, J617, SLH3, 7P221, C1505, F11x0, F1440, F3941, G8523, J6708, J6710, U60H3, ZV113, ZV4KC, ZV57A, ZV6D6<br>Except: 9k13, 9k15, 9k16, 9k17, 9k18, 9k19
Common mental disorder	Eu3, Eu4, E20, E26, E28, E29, E2B, E112, E113, E118, E135, E120, E278<br>Except: Eu30, Eu31, E20y (except: E20y0, E20yz), Eu323, Eu325, Eu326, Eu327, Eu328, Eu329, Eu32A, Eu32B, Eu333, Eu460, Eu46y, E1124, E1134, E2922
Depression and anxiety	1BQ, 1BT, 1BU, E2B, 1B12, 1B13, 1B17, 1B1U, 2257, 2258, 2259, E112, E113, E118, E135, E200, E204, E291, Eu32, Eu33, Eu41, R2y2, Eu341
Drugs misuse	1V, du, 13c, 1TE, 1TF, 8AA, 8FB, 8Hq, 9G2, 9HC, 9k5, E02, E24, E25, Eu1, djc, 146C, 1P30, 1P31, 677T, 8B23, 8BAW, 8BAX, 8BAc, 8CR9, 8H7x, 8HHL, 8HHd, 8HHe, 8Hh1, 8HkF, 8Hl5, 8Hl6, 8I2N, 9N6a, 9N6b, 9NN1, 9NdN, 9No5, L183, R10B, SL97, T800, dj36, dj37, dj38, dj3D, dj3E, dj3F, dj3G, dj3u, dj3v, 7P220, 9N1yJ, ZV6D7<br>Except: du1, du3, du6, du7, du8, du9, duA, duB, 13cP, 1V23, 1V25, 1V26, 1V50, 1V60, 1V61, 1V62, 1V63, 8FB0, 9HC0, 9HC1, 9HC5, 9HC8, E023, E250, E251, Eu10, du24, Eu170, Eu171, Eu172, Eu173, Eu174, Eu175, Eu176, Eu177, Eu17y, Eu17z, SL971
Injury and poisoning	S, T, U
Mental health	E
Self-harm	U2, SL, U720, TK601<br>Except: U209
Prescription of opiates	8B23, 8B2M, 8B2N, 8B2P, 8B2Q, 8B2R, 8BE, a81, cg1, cg3, cg5, cg7, dia, dib, dic, did6, dj, j28O, j28V, j2pT, o4, oa2N, oa2J<br>Except: dia8A-diaF, diaH, diaM, diaN, diaQ, diaR, diaV-diaZ, diaa, diac, diaf, diag, diaj, dial, diau. diay, diaz, dib1, dib2, dib4-dib7, dibC, dibL, dibN, dibO, dibP, dibT, dibU, dibV, dibY, dibZ, dibi, dibk, dibl, dibm, dibq, dibr, diby, dic1, dic2, dic6, dicB-dicI, dicP, dicQ, dicT-dicX, dicZ, dicw, djm, o43, o4a, cg7c, dj3K, dj3L, dj3M, djkb, djkc, djkd, djyJ, djyK, djyL, o4e4-o4e8
Prescription of psychotropics	d1, d2, d4, d5-d9, da, gde<br>Except: d19, d1e, d4y, da8, d4v9, drvR, d4w9, d4wR, d915

Table A3: Comparison between error rates of Table 1 using the corrected resampled t-test based on the average over sorted runs of 10x10 k-folds.

	Mean	SE	*z*	*P-value*	*Q-value*
nn10-10 vs					
nn50-50	-0.046	0.030	-0.885	.188	.232
nn100-100	-0.049	0.032	-0.912	.181	.232
nn50	-0.305	0.183	-2.364	.009	.021
nn100	-0.322	0.204	-2.365	.009	.021
nn10	-0.335	0.194	-2.522	.006	.017
nn0	-2.107	1.063	-6.776	<.001	<.001
nn50-50 vs					
nn100-100	-0.003	0.020	-0.070	.472	.472
nn50	-0.259	0.195	-1.946	.026	.036
nn100	-0.277	0.180	-2.160	.015	.025
nn10	-0.289	0.198	-2.154	.016	.025
nn0	-2.061	1.085	-6.562	<.001	<.001
nn100-100 vs					
nn50	-0.256	0.166	-2.085	.019	.028
nn100	-0.274	0.176	-2.161	.015	.025
nn10	-0.286	0.179	-2.245	.012	.025
nn0	-2.058	1.105	-6.493	<.001	<.001
nn50 vs					
nn100	-0.017	0.008	-0.664	.253	.280
nn10	-0.030	0.017	-0.773	.220	.256
nn0	-1.802	0.902	-6.291	<.001	<.001
nn100 vs					
nn10	-0.013	0.017	-0.323	.373	.392
nn0	-1.784	0.892	-6.266	<.001	<.001
nn10 vs					
nn0	-1.772	0.858	-6.344	<.001	<.001

* **Mean** difference, standard error (**SE**), **z**-values, **p-values** and FDR-BH corrected values (**q-values**).

Table A4: Comparison between AUROC of Table 1 using the corrected resampled t-test based on the average over sorted runs of 10x10 k-folds.

	Mean	SE	*z*	*p-value*	*q-value*
nn10-10 vs					
nn50-50	0.000	0.000	2.075	.019	.023
nn100-100	0.001	0.000	2.688	.004	.005
nn50	0.005	0.000	12.222	<.001	<.001
nn100	0.005	0.000	12.355	<.001	<.001
nn10	0.005	0.000	12.581	<.001	<.001
nn0	0.018	0.018	15.116	<.001	<.001
nn50-50 vs					
nn100-100	0.000	0.000	1.272	.102	.107
nn50	0.005	0.000	11.349	<.001	<.001
nn100	0.005	0.000	22.354	<.001	<.001
nn10	0.005	0.000	11.322	<.001	<.001
nn0	0.018	0.018	15.099	<.001	<.001
nn100-100 vs					
nn50	0.004	0.000	11.146	<.001	<.001
nn100	0.004	0.000	11.186	<.001	<.001
nn10	0.005	0.000	11.322	<.001	<.001
nn0	0.017	0.017	15.080	<.001	<.001
nn50 vs					
nn100	-0.000	0.000	-0.649	.258	.258
nn10	0.000	0.000	1.320	.093	.103
nn0	0.013	0.013	14.485	<.001	<.001
nn100 vs					
nn10	0.000	0.000	1.578	.057	.067
nn0	0.013	0.000	14.575	<.001	<.001
nn10 vs					
nn0	0.013	0.000	13.936	<.001	<.001

* **Mean** difference, standard error (**SE**), **z**-values, **p-values** and FDR-BH corrected values (**q-values**).

Table A5: Distribution of the 1M time-frame factors (individuals presenting each factor) across cases, control and risk scores.

Factor	Controls N % [95% CI]	Cases N % [95% CI]	VLR N % [95% CI]	LR N % [95% CI]	MLR N % [95% CI]	MHR N % [95% CI]	HR N % [95% CI]	VHR N % [95% CI]
**Total**	**52080**	**2604**	**70**	**25744**	**17818**	**6011**	**3675**	**1366**
Depression & anxiety	254 52.16 [48.43, 55.86]	233 47.84 [44.14, 51.57]	0 0.00 [0.00, 0.55]	3 0.62 [0.25, 1.53]	17 3.49 [2.36, 5.14]	60 12.32 [10.08, 14.98]	149 30.60 [27.28, 34.13]	258 52.98 [49.25, 56.67]
Other common mental disorders	39 60.00 [49.80, 69.40]	26 40.00 [30.60, 50.20]	0 0.00 [0.00, 4.00]	13 20.00 [13.11, 29.28]	13 20.00 [13.11, 29.28]	8 12.31 [7.08, 20.55]	17 26.15 [18.27, 35.94]	14 21.54 [14.38, 30.97]
Other mental health	71 76.34 [68.42, 82.78]	22 23.66 [17.22, 31.58]	0 0.00 [0.00, 2.83]	21 22.58 [16.28, 30.43]	26 27.96 [21.01, 36.15]	8 8.60 [4.91, 14.63]	24 25.81 [19.10, 33.88]	14 15.05 [9.95, 22.13]
Unintentional injury&poisoning	400 79.84 [76.74, 82.62]	101 20.16 [17.38, 23.26]	0 0.00 [0.00, 0.54]	31 6.19 [4.64, 8.20]	207 41.32 [37.75, 44.97]	98 19.56 [16.81, 22.64]	84 16.77 [14.20, 19.69]	81 16.17 [13.65, 19.05]
Self-harm	18 13.95 [9.67, 19.72]	111 86.05 [80.28, 90.33]	0 0.00 [0.00, 2.05]	0 0.00 [0.00, 2.05]	6 4.65 [2.42, 8.74]	10 7.75 [4.69, 12.55]	37 28.68 [22.62, 35.62]	76 58.91 [51.68, 65.79]
Alcohol misuse	46 45.54 [37.62, 53.71]	55 54.46 [46.29, 62.38]	0 0.00 [0.00, 2.61]	1 0.99 [0.22, 4.32]	8 7.92 [4.52, 13.52]	14 13.86 [9.14, 20.46]	16 15.84 [10.77, 22.70]	62 61.39 [53.22, 68.96]
Drugs misuse	41 60.29 [50.32, 69.48]	27 39.71 [30.52, 49.68]	0 0.00 [0.00, 3.83]	2 2.94 [0.98, 8.51]	6 8.82 [4.63, 16.17]	7 10.29 [5.68, 17.95]	9 13.24 [7.87, 21.42]	44 64.71 [54.78, 73.51]
Possible maltreatment	12 70.59 [50.64, 84.88]	5 29.41 [15.12, 49.36]	0 0.00 [0.00, 13.73]	6 35.29 [19.49, 55.14]	3 17.65 [7.28, 36.90]	2 11.76 [3.97, 30.06]	3 17.65 [7.28, 36.90]	3 17.65 [7.28, 36.90]
Non-physical sleep disorder	48 67.61 [57.97, 75.95]	23 32.39 [24.05, 42.03]	0 0.00 [0.00, 3.67]	7 9.86 [5.43, 17.23]	16 22.54 [15.48, 31.61]	19 26.76 [19.09, 36.14]	18 25.35 [17.87, 34.64]	11 15.49 [9.71, 23.81]
Physical sleep disorder	116 98.31 [95.01, 99.44]	<5 <4.24 [2.08, 8.45]	<5 <4.24 [2.08, 8.45]	82 69.49 [62.15, 75.96]	14 11.86 [7.80, 17.64]	11 9.32 [5.79, 14.68]	6 5.08 [2.65, 9.53]	<5 <4.24 [2.08, 8.45]
Prescription of opiates	3260 92.22 [91.45, 92.93]	275 7.78 [7.07, 8.55]	11 0.31 [0.19, 0.51]	1293 36.58 [35.26, 37.92]	712 20.14 [19.05, 21.27]	598 16.92 [15.90, 17.98]	669 18.93 [17.87, 20.03]	252 7.13 [6.45, 7.87]
Prescription of psychotropics	4266 82.23 [81.34, 83.08]	922 17.77 [16.92, 18.66]	0 0.00 [0.00, 0.05]	4 0.08 [0.03, 0.17]	147 2.83 [2.48, 3.24]	1177 22.69 [21.74, 23.66]	2745 52.91 [51.77, 54.05]	1115 21.49 [20.57, 22.44]
Other events	22029 94.17 [93.92, 94.42]	1363 5.83 [5.58, 6.08]	26 0.11 [0.08, 0.15]	10100 43.18 [42.65, 43.71]	7848 33.55 [33.04, 34.06]	2319 9.91 [9.60, 10.24]	2202 9.41 [9.10, 9.73]	897 3.83 [3.63, 4.05]
GP events	24534 93.67 [93.41, 93.91]	1659 6.33 [6.09, 6.59]	28 0.11 [0.08, 0.15]	10617 40.53 [40.04, 41.03]	8125 31.02 [30.55, 31.49]	2905 11.09 [10.78, 11.41]	3260 12.45 [12.11, 12.79]	1258 4.80 [4.59, 5.02]
Hospital admissions	943 81.08 [79.12, 82.90]	220 18.92 [17.10, 20.88]	0 0.00 [0.00, 0.23]	107 9.20 [7.90, 10.69]	405 34.82 [32.56, 37.15]	234 20.12 [18.26, 22.12]	213 18.31 [16.52, 20.25]	204 17.54 [15.78, 19.45]

Table A6: Distribution of the 6M time-frame factors (individuals presenting each factor) across cases, control and risk scores.

Factor	Controls N % [95% CI]	Cases N % [95% CI]	VLR N % [95% CI]	LR N % [95% CI]	MLR N % [95% CI]	MHR N % [95% CI]	HR N % [95% CI]	VHR N % [95% CI]
**Total**	**52080**	**2604**	**70**	**25744**	**17818**	**6011**	**3675**	**1366**
Depression & anxiety	1000 71.53 [69.51, 73.47]	398 28.47 [26.53, 30.49]	0 0.00 [0.00, 0.19]	12 0.86 [0.54, 1.37]	87 6.22 [5.24, 7.37]	245 17.53 [15.92, 19.26]	490 35.05 [32.98, 37.18]	564 40.34 [38.21, 42.52]
Other common mental disorders	148 73.27 [67.86, 78.06]	54 26.73 [21.94, 32.14]	0 0.00 [0.00, 1.32]	28 13.86 [10.34, 18.34]	40 19.80 [15.60, 24.80]	36 17.82 [13.83, 22.67]	41 20.30 [16.05, 25.33]	57 28.22 [23.32, 33.69]
Other mental health	281 87.00 [83.61, 89.77]	42 13.00 [10.23, 16.39]	1 0.31 [0.07, 1.38]	91 28.17 [24.25, 32.46]	92 28.48 [24.54, 32.78]	41 12.69 [9.95, 16.05]	62 19.20 [15.85, 23.05]	36 11.15 [8.58, 14.35]
Unintentional injury&poisoning	1824 90.03 [88.88, 91.07]	202 9.97 [8.93, 11.12]	0 0.00 [0.00, 0.13]	514 25.37 [23.81, 26.99]	839 41.41 [39.62, 43.22]	265 13.08 [11.90, 14.36]	237 11.70 [10.57, 12.92]	171 8.44 [7.48, 9.51]
Self-harm	60 27.78 [23.06, 33.04]	156 72.22 [66.96, 76.94]	0 0.00 [0.00, 1.24]	1 0.46 [0.10, 2.05]	9 4.17 [2.44, 7.03]	21 9.72 [6.89, 13.55]	42 19.44 [15.40, 24.24]	143 66.20 [60.74, 71.27]
Alcohol misuse	163 57.80 [52.91, 62.54]	119 42.20 [37.46, 47.09]	0 0.00 [0.00, 0.95]	11 3.90 [2.40, 6.28]	36 12.77 [9.85, 16.39]	40 14.18 [11.11, 17.94]	56 19.86 [16.25, 24.04]	139 49.29 [44.42, 54.17]
Drugs misuse	109 63.37 [57.17, 69.16]	63 36.63 [30.84, 42.83]	0 0.00 [0.00, 1.55]	11 6.40 [3.95, 10.19]	25 14.53 [10.66, 19.50]	24 13.95 [10.16, 18.86]	31 18.02 [13.71, 23.33]	81 47.09 [40.93, 53.35]
Possible maltreatment	43 72.88 [62.52, 81.24]	16 27.12 [18.76, 37.48]	0 0.00 [0.00, 4.38]	20 33.90 [24.67, 44.54]	13 22.03 [14.50, 32.03]	5 8.47 [4.19, 16.40]	4 6.78 [3.08, 14.27]	17 28.81 [20.21, 39.27]
Non-physical sleep disorder	216 79.70 [75.40, 83.42]	55 20.30 [16.58, 24.60]	2 0.74 [0.24, 2.21]	29 10.70 [7.99, 14.19]	63 23.25 [19.30, 27.72]	51 18.82 [15.23, 23.03]	83 30.63 [26.23, 35.41]	43 15.87 [12.56, 19.85]
Physical sleep disorder	573 95.98 [94.44, 97.11]	24 4.02 [2.89, 5.56]	27 4.52 [3.32, 6.14]	362 60.64 [57.31, 63.87]	92 15.41 [13.14, 18.00]	67 11.22 [9.27, 13.53]	36 6.03 [4.62, 7.84]	13 2.18 [1.39, 3.40]
Prescription of opiates	6106 93.15 [92.62, 93.65]	449 6.85 [6.35, 7.38]	29 0.44 [0.33, 0.60]	2645 40.35 [39.36, 41.35]	1532 23.37 [22.52, 24.24]	968 14.77 [14.06, 15.50]	1017 15.51 [14.79, 16.26]	364 5.55 [5.11, 6.04]
Prescription of psychotropics	6188 85.12 [84.42, 85.79]	1082 14.88 [14.21, 15.58]	0 0.00 [0.00, 0.04]	58 0.80 [0.64, 0.99]	849 11.68 [11.07, 12.31]	2053 28.24 [27.38, 29.12]	3090 42.50 [41.55, 43.46]	1220 16.78 [16.07, 17.51]
Other events	35755 94.97 [94.78, 95.15]	1893 5.03 [4.85, 5.22]	66 0.18 [0.14, 0.21]	20141 53.50 [53.08, 53.92]	9699 25.76 [25.39, 26.13]	3324 8.83 [8.59, 9.07]	3205 8.51 [8.28, 8.75]	1213 3.22 [3.08, 3.38]
GP events	36763 94.93 [94.74, 95.11]	1965 5.07 [4.89, 5.26]	69 0.18 [0.15, 0.22]	20472 52.86 [52.44, 53.28]	9935 25.65 [25.29, 26.02]	3540 9.14 [8.90, 9.38]	3421 8.83 [8.60, 9.07]	1291 3.33 [3.19, 3.49]
Hospital admissions	3499 87.65 [86.77, 88.48]	493 12.35 [11.52, 13.23]	0 0.00 [0.00, 0.07]	515 12.90 [12.05, 13.80]	1659 41.56 [40.28, 42.85]	674 16.88 [15.93, 17.88]	659 16.51 [15.56, 17.50]	485 12.15 [11.32, 13.03]

Table A7: Distribution of the 1Y time-frame factors (individuals presenting each factor) across cases, control and risk scores.

**Factor**	**Controls** N % [95% CI]	**Cases** N % [95% CI]	**VLR** N % [95% CI]	**LR** N % [95% CI]	**MLR** N % [95% CI]	**MHR** N % [95% CI]	**HR** N % [95% CI]	**VHR** N % [95% CI]
**Total**	**52080**	**2604**	**70**	**25744**	**17818**	**6011**	**3675**	**1366**
Depression & anxiety	1185 79.58 [77.81, 81.25]	304 20.42 [18.75, 22.19]	0 0.00 [0.00, 0.18]	71 4.77 [3.94, 5.76]	247 16.59 [15.06, 18.23]	294 19.74 [18.10, 21.50]	457 30.69 [28.76, 32.69]	420 28.21 [26.33, 30.16]
Other common mental disorders	183 80.97 [76.32, 84.89]	43 19.03 [15.11, 23.68]	0 0.00 [0.00, 1.18]	62 27.43 [22.84, 32.56]	48 21.24 [17.12, 26.04]	35 15.49 [11.94, 19.85]	40 17.70 [13.91, 22.25]	41 18.14 [14.31, 22.73]
Other mental health	341 88.34 [85.38, 90.77]	45 11.66 [9.23, 14.62]	2 0.52 [0.17, 1.55]	134 34.72 [30.85, 38.79]	102 26.42 [22.91, 30.27]	46 11.92 [9.47, 14.90]	66 17.10 [14.18, 20.48]	36 9.33 [7.17, 12.05]
Unintentional injury&poisoning	2113 92.11 [91.13, 92.99]	181 7.89 [7.01, 8.87]	6 0.26 [0.14, 0.50]	993 43.29 [41.59, 45.00]	690 30.08 [28.53, 31.68]	224 9.76 [8.79, 10.83]	239 10.42 [9.42, 11.51]	142 6.19 [5.41, 7.07]
Self-harm	53 28.80 [23.65, 34.57]	131 71.20 [65.43, 76.35]	0 0.00 [0.00, 1.45]	2 1.09 [0.36, 3.23]	21 11.41 [8.10, 15.84]	17 9.24 [6.29, 13.37]	33 17.93 [13.76, 23.04]	111 60.33 [54.29, 66.07]
Alcohol misuse	171 64.04 [59.10, 68.71]	96 35.96 [31.29, 40.90]	0 0.00 [0.00, 1.00]	12 4.49 [2.83, 7.08]	42 15.73 [12.41, 19.74]	39 14.61 [11.41, 18.52]	52 19.48 [15.80, 23.76]	122 45.69 [40.75, 50.73]
Drugs misuse	135 68.18 [62.52, 73.35]	63 31.82 [26.65, 37.48]	0 0.00 [0.00, 1.35]	13 6.57 [4.22, 10.09]	29 14.65 [10.99, 19.26]	30 15.15 [11.43, 19.81]	38 19.19 [15.02, 24.20]	88 44.44 [38.75, 50.29]
Possible maltreatment	61 77.22 [68.63, 84.00]	18 22.78 [16.00, 31.37]	0 0.00 [0.00, 3.31]	24 30.38 [22.64, 39.42]	25 31.65 [23.77, 40.74]	10 12.66 [7.72, 20.07]	9 11.39 [6.75, 18.59]	11 13.92 [8.71, 21.53]
Non-physical sleep disorder	235 86.72 [82.96, 89.75]	36 13.28 [10.25, 17.04]	1 0.37 [0.08, 1.64]	58 21.40 [17.60, 25.77]	58 21.40 [17.60, 25.77]	57 21.03 [17.26, 25.38]	65 23.99 [19.99, 28.50]	32 11.81 [8.96, 15.42]
Physical sleep disorder	671 95.45 [93.97, 96.58]	32 4.55 [3.42, 6.03]	20 2.84 [1.98, 4.07]	438 62.30 [59.26, 65.26]	114 16.22 [14.06, 18.63]	67 9.53 [7.86, 11.51]	47 6.69 [5.30, 8.41]	17 2.42 [1.63, 3.57]
Prescription of opiates	6399 93.44 [92.93, 93.92]	449 6.56 [6.08, 7.07]	30 0.44 [0.32, 0.59]	2898 42.32 [41.34, 43.30]	1548 22.61 [21.78, 23.45]	1002 14.63 [13.94, 15.35]	1019 14.88 [14.19, 15.60]	351 5.13 [4.70, 5.58]
Prescription of psychotropics	6300 87.05 [86.39, 87.69]	937 12.95 [12.31, 13.61]	0 0.00 [0.00, 0.04]	374 5.17 [4.76, 5.61]	1113 15.38 [14.69, 16.09]	1909 26.38 [25.54, 27.24]	2782 38.44 [37.51, 39.39]	1059 14.63 [13.96, 15.33]
Other events	37211 95.22 [95.04, 95.40]	1866 4.78 [4.60, 4.96]	70 0.18 [0.15, 0.22]	22151 56.69 [56.27, 57.10]	9348 23.92 [23.57, 24.28]	3161 8.09 [7.87, 8.32]	3173 8.12 [7.90, 8.35]	1174 3.00 [2.87, 3.15]
GP events	38127 95.19 [95.01, 95.36]	1926 4.81 [4.64, 4.99]	70 0.17 [0.14, 0.21]	22518 56.22 [55.81, 56.63]	9557 23.86 [23.51, 24.21]	3308 8.26 [8.04, 8.49]	3349 8.36 [8.14, 8.59]	1251 3.12 [2.98, 3.27]
Hospital admissions	3895 89.42 [88.63, 90.16]	461 10.58 [9.84, 11.37]	2 0.05 [0.02, 0.14]	889 20.41 [19.42, 21.43]	1743 40.01 [38.80, 41.24]	632 14.51 [13.65, 15.41]	647 14.85 [13.99, 15.76]	443 10.17 [9.44, 10.95]

Table A8: Distribution of the 5Y time-frame factors (individuals presenting each factor) across cases, control and risk scores.

Factor	Controls	Cases	VLR	LR	MLR	MHR	HR	VHR
	N % [95% CI]	N % [95% CI]	N % [95% CI]	N % [95% CI]	N % [95% CI]	N % [95% CI]	N % [95% CI]	N % [95% CI]
**Total**	**52080**	**2604**	**70**	**25744**	**17818**	**6011**	**3675**	**1366**
Depression & anxiety	5645 86.87 [86.17, 87.55]	853 13.13 [12.45, 13.83]	0 0.00 [0.00, 0.04]	403 6.20 [5.73, 6.71]	2016 31.02 [30.09, 31.98]	1333 20.51 [19.70, 21.35]	1765 27.16 [26.26, 28.08]	981 15.10 [14.38, 15.84]
Other common mental disorders	1254 87.45 [85.94, 88.82]	180 12.55 [11.18, 14.06]	7 0.49 [0.26, 0.90]	436 30.40 [28.44, 32.44]	339 23.64 [21.85, 25.53]	205 14.30 [12.84, 15.88]	261 18.20 [16.59, 19.94]	186 12.97 [11.58, 14.50]
Other mental health	1800 90.18 [89.03, 91.22]	196 9.82 [8.78, 10.97]	0 0.00 [0.00, 0.14]	582 29.16 [27.51, 30.86]	650 32.57 [30.86, 34.31]	222 11.12 [10.02, 12.33]	341 17.08 [15.74, 18.51]	201 10.07 [9.02, 11.23]
Unintentional injury&poisoning	12793 93.75 [93.40, 94.08]	853 6.25 [5.92, 6.60]	14 0.10 [0.07, 0.16]	5310 38.91 [38.23, 39.60]	5086 37.27 [36.59, 37.95]	1292 9.47 [9.06, 9.89]	1269 9.30 [8.90, 9.72]	675 4.95 [4.65, 5.26]
Self-harm	441 55.68 [52.76, 58.56]	351 44.32 [41.44, 47.24]	0 0.00 [0.00, 0.34]	1 0.13 [0.03, 0.56]	39 4.92 [3.81, 6.35]	126 15.91 [13.89, 18.16]	208 26.26 [23.77, 28.91]	418 52.78 [49.86, 55.68]
Alcohol misuse	772 73.95 [71.65, 76.12]	272 26.05 [23.88, 28.35]	0 0.00 [0.00, 0.26]	9 0.86 [0.50, 1.48]	175 16.76 [14.95, 18.75]	251 24.04 [21.94, 26.28]	226 21.65 [19.63, 23.82]	383 36.69 [34.27, 39.17]
Drugs misuse	508 71.25 [68.38, 73.95]	205 28.75 [26.05, 31.62]	0 0.00 [0.00, 0.38]	13 1.82 [1.16, 2.85]	98 13.74 [11.76, 16.00]	145 20.34 [17.97, 22.93]	160 22.44 [19.98, 25.11]	297 41.65 [38.66, 44.72]
Possible maltreatment	505 85.16 [82.60, 87.40]	88 14.84 [12.60, 17.40]	0 0.00 [0.00, 0.45]	92 15.51 [13.23, 18.12]	235 39.63 [36.38, 42.97]	102 17.20 [14.80, 19.90]	91 15.35 [13.07, 17.94]	73 12.31 [10.26, 14.70]
Non-physical sleep disorder	1439 89.16 [87.82, 90.37]	175 10.84 [9.63, 12.18]	2 0.12 [0.04, 0.37]	408 25.28 [23.54, 27.10]	426 26.39 [24.63, 28.24]	255 15.80 [14.36, 17.35]	341 21.13 [19.51, 22.85]	182 11.28 [10.05, 12.64]
Physical sleep disorder	2070 95.79 [95.02, 96.44]	91 4.21 [3.56, 4.98]	51 2.36 [1.88, 2.96]	1346 62.29 [60.56, 63.98]	326 15.09 [13.86, 16.40]	211 9.76 [8.76, 10.87]	167 7.73 [6.84, 8.73]	60 2.78 [2.25, 3.42]
Prescription of opiates	15742 94.59 [94.29, 94.87]	901 5.41 [5.13, 5.71]	63 0.38 [0.31, 0.47]	8672 52.11 [51.47, 52.74]	3674 22.08 [21.55, 22.61]	1776 10.67 [10.28, 11.07]	1810 10.88 [10.48, 11.28]	648 3.89 [3.65, 4.15]
Prescription of psychotropics	11375 90.12 [89.67, 90.55]	1247 9.88 [9.45, 10.33]	0 0.00 [0.00, 0.02]	1716 13.60 [13.10, 14.10]	4007 31.75 [31.07, 32.43]	2635 20.88 [20.29, 21.48]	3064 24.28 [23.65, 24.91]	1200 9.51 [9.09, 9.95]
Other events	49544 95.40 [95.25, 95.55]	2387 4.60 [4.45, 4.75]	70 0.13 [0.11, 0.16]	25702 49.49 [49.13, 49.85]	17342 33.39 [33.05, 33.74]	3885 7.48 [7.29, 7.67]	3602 6.94 [6.75, 7.12]	1330 2.56 [2.45, 2.68]
GP events	49436 95.58 [95.43, 95.73]	2286 4.42 [4.27, 4.57]	70 0.14 [0.11, 0.16]	25744 49.77 [49.41, 50.14]	17434 33.71 [33.37, 34.05]	3672 7.10 [6.92, 7.29]	3499 6.77 [6.59, 6.95]	1303 2.52 [2.41, 2.64]
Hospital admissions	16988 92.91 [92.59, 93.21]	1297 7.09 [6.79, 7.41]	6 0.03 [0.02, 0.06]	5665 30.98 [30.42, 31.55]	7178 39.26 [38.66, 39.85]	2208 12.08 [11.68, 12.48]	2205 12.06 [11.67, 12.46]	1023 5.59 [5.32, 5.88]

Table A9: Incidence of the 1M time-frame factors (individuals presenting each factor) for cases, controls and risk scores.

Factor	Controls	Cases	VLR	LR	MLR	MHR	HR	VHR
	N % [95% CI]	N % [95% CI]	N % [95% CI]	N % [95% CI]	N % [95% CI]	N % [95% CI]	N % [95% CI]	N % [95% CI]
**Total**	**52080**	**2604**	**70**	**25744**	**17818**	**6011**	**3675**	**1366**
Depression & anxiety	254 0.49 [0.44, 0.54]	233 8.95 [8.07, 9.91]	0 0.00 [0.00, 3.72]	3 0.01 [0.00, 0.03]	17 0.10 [0.06, 0.14]	60 1.00 [0.81, 1.23]	149 4.05 [3.55, 4.62]	258 18.89 [17.21, 20.69]
Other common mental disorders	39 0.07 [0.06, 0.10]	26 1.00 [0.73, 1.37]	0 0.00 [0.00, 3.72]	13 0.05 [0.03, 0.08]	13 0.07 [0.05, 0.11]	8 0.13 [0.08, 0.24]	17 0.46 [0.31, 0.69]	14 1.02 [0.66, 1.58]
Other mental health	71 0.14 [0.11, 0.17]	22 0.84 [0.60, 1.20]	0 0.00 [0.00, 3.72]	21 0.08 [0.06, 0.12]	26 0.15 [0.11, 0.20]	8 0.13 [0.08, 0.24]	24 0.65 [0.47, 0.91]	14 1.02 [0.66, 1.58]
Unintentional injury&poisoning	400 0.77 [0.71, 0.83]	101 3.88 [3.30, 4.55]	0 0.00 [0.00, 3.72]	31 0.12 [0.09, 0.16]	207 1.16 [1.04, 1.30]	98 1.63 [1.38, 1.92]	84 2.29 [1.91, 2.73]	81 5.93 [4.96, 7.07]
Self-harm	18 0.03 [0.02, 0.05]	111 4.26 [3.66, 4.96]	0 0.00 [0.00, 3.72]	0 0.00 [0.00, 0.01]	6 0.03 [0.02, 0.07]	10 0.17 [0.10, 0.28]	37 1.01 [0.77, 1.32]	76 5.56 [4.63, 6.67]
Alcohol misuse	46 0.09 [0.07, 0.11]	55 2.11 [1.70, 2.63]	0 0.00 [0.00, 3.72]	1 0.00 [0.00, 0.02]	8 0.04 [0.03, 0.08]	14 0.23 [0.15, 0.36]	16 0.44 [0.29, 0.65]	62 4.54 [3.70, 5.56]
Drugs misuse	41 0.08 [0.06, 0.10]	27 1.04 [0.76, 1.42]	0 0.00 [0.00, 3.72]	2 0.01 [0.00, 0.02]	6 0.03 [0.02, 0.07]	7 0.12 [0.06, 0.21]	9 0.24 [0.14, 0.42]	44 3.22 [2.52, 4.10]
Possible maltreatment	12 0.02 [0.01, 0.04]	5 0.19 [0.09, 0.39]	0 0.00 [0.00, 3.72]	6 0.02 [0.01, 0.05]	3 0.02 [0.01, 0.04]	2 0.03 [0.01, 0.10]	3 0.08 [0.03, 0.20]	3 0.22 [0.09, 0.55]
Non-physical sleep disorder	48 0.09 [0.07, 0.12]	23 0.88 [0.63, 1.24]	0 0.00 [0.00, 3.72]	7 0.03 [0.01, 0.05]	16 0.09 [0.06, 0.14]	19 0.32 [0.22, 0.46]	18 0.49 [0.33, 0.72]	11 0.81 [0.49, 1.31]
Physical sleep disorder	116 0.22 [0.19, 0.26]	<5 <0.19 [0.09, 0.39]	<5 <7.14 [3.52, 13.96]	82 0.32 [0.27, 0.38]	14 0.08 [0.05, 0.12]	11 0.18 [0.11, 0.30]	6 0.16 [0.08, 0.32]	<5 <0.37 [0.18, 0.75]
Prescription of opiates	3260 6.26 [6.09, 6.44]	275 10.56 [9.61, 11.59]	11 15.71 [9.85, 24.13]	1293 5.02 [4.80, 5.25]	712 4.00 [3.76, 4.24]	598 9.95 [9.33, 10.60]	669 18.20 [17.18, 19.27]	252 18.45 [16.78, 20.24]
Prescription of psychotropics	4266 8.19 [8.00, 8.39]	922 35.41 [33.88, 36.96]	0 0.00 [0.00, 3.72]	4 0.02 [0.01, 0.03]	147 0.83 [0.72, 0.94]	1177 19.58 [18.75, 20.44]	2745 74.69 [73.50, 75.86]	1115 81.63 [79.84, 83.29]
Other events	22029 42.30 [41.94, 42.65]	1363 52.34 [50.73, 53.95]	26 37.14 [28.29, 46.95	10100 39.23 [38.73, 39.73]	7848 44.05 [43.43, 44.66]	2319 38.58 [37.55, 39.62]	2202 59.92 [58.58, 61.24]	897 65.67 [63.52, 67.75]
GP events	24534 47.11 [46.75, 47.47]	1659 63.71 [62.15, 65.24]	28 40.00 [30.91, 49.83	10617 41.24 [40.74, 41.75]	8125 45.60 [44.99, 46.21]	2905 48.33 [47.27, 49.39]	3260 88.71 [87.82, 89.54]	1258 92.09 [90.81, 93.21]
Hospital admissions	943 1.81 [1.72, 1.91]	220 8.45 [7.59, 9.39]	0 0.00 [0.00, 3.72]	107 0.42 [0.35, 0.49]	405 2.27 [2.10, 2.46]	234 3.89 [3.50, 4.32]	213 5.80 [5.19, 6.46]	204 14.93 [13.42, 16.59]

Table A10: Incidence of the 6M time-frame factors (individuals presenting each factor) for cases, controls and risk scores.

Factor	Controls<br>N % [95% CI]	Cases<br>N % [95% CI]	VLR<br>N % [95% CI]	LR<br>N % [95% CI]	MLR<br>N % [95% CI]	MHR<br>N % [95% CI]	HR<br>N % [95% CI]	VHR<br>N % [95% CI]
**Total**	**52080**	**2604**	**70**	**25744**	**17818**	**6011**	**3675**	**1366**
Depression & anxiety	1000 1.92 [1.82, 2.02]	398 15.28 [14.16, 16.48]	0 0.00 [0.00, 3.72]	12 0.05 [0.03, 0.07]	87 0.49 [0.41, 0.58]	245 4.08 [3.68, 4.52]	490 13.33 [12.44, 14.28]	564 41.29 [39.12, 43.49]
Other common mental disorders	148 0.28 [0.25, 0.33]	54 2.07 [1.66, 2.59]	0 0.00 [0.00, 3.72]	28 0.11 [0.08, 0.15]	40 0.22 [0.17, 0.29]	36 0.60 [0.46, 0.79]	41 1.12 [0.86, 1.44]	57 4.17 [3.37, 5.16]
Other mental health	281 0.54 [0.49, 0.59]	42 1.61 [1.25, 2.07]	1 1.43 [0.32, 6.15]	91 0.35 [0.30, 0.42]	92 0.52 [0.44, 0.61]	41 0.68 [0.53, 0.88]	62 1.69 [1.37, 2.07]	36 2.64 [2.01, 3.45]
Unintentional injury&poisoning	1824 3.50 [3.37, 3.64]	202 7.76 [6.94, 8.66]	0 0.00 [0.00, 3.72]	514 2.00 [1.86, 2.15]	839 4.71 [4.45, 4.98]	265 4.41 [3.99, 4.87]	237 6.45 [5.81, 7.15]	171 12.52 [11.12, 14.07]
Self-harm	60 0.12 [0.09, 0.14]	156 5.99 [5.27, 6.80]	0 0.00 [0.00, 3.72]	1 0.00 [0.00, 0.02]	9 0.05 [0.03, 0.09]	21 0.35 [0.24, 0.50]	42 1.14 [0.89, 1.47]	143 10.47 [9.18, 11.91]
Alcohol misuse	163 0.31 [0.28, 0.36]	119 4.57 [3.94, 5.29]	0 0.00 [0.00, 3.72]	11 0.04 [0.03, 0.07]	36 0.20 [0.15, 0.27]	40 0.67 [0.51, 0.86]	56 1.52 [1.23, 1.89]	139 10.18 [8.91, 11.60]
Drugs misuse	109 0.21 [0.18, 0.24]	63 2.42 [1.97, 2.97]	0 0.00 [0.00, 3.72]	11 0.04 [0.03, 0.07]	25 0.14 [0.10, 0.19]	24 0.40 [0.29, 0.56]	31 0.84 [0.63, 1.13]	81 5.93 [4.96, 7.07]
Possible maltreatment	43 0.08 [0.06, 0.11]	16 0.61 [0.41, 0.92]	0 0.00 [0.00, 3.72]	20 0.08 [0.05, 0.11]	13 0.07 [0.05, 0.11]	5 0.08 [0.04, 0.17]	4 0.11 [0.05, 0.24]	17 1.24 [0.84, 1.84]
Non-physical sleep disorder	216 0.41 [0.37, 0.46]	55 2.11 [1.70, 2.63]	2 2.86 [0.95, 8.27]	29 0.11 [0.08, 0.15]	63 0.35 [0.29, 0.43]	51 0.85 [0.67, 1.07]	83 2.26 [1.89, 2.70]	43 3.15 [2.46, 4.02]
Physical sleep disorder	573 1.10 [1.03, 1.18]	24 0.92 [0.66, 1.28]	27 38.57 [29.60, 48.40]	362 1.41 [1.29, 1.53]	92 0.52 [0.44, 0.61]	67 1.11 [0.91, 1.36]	36 0.98 [0.75, 1.29]	13 0.95 [0.61, 1.49]
Prescription of opiates	6106 11.72 [11.49, 11.96]	449 17.24 [16.06, 18.49]	29 41.43 [32.24, 51.26]	2645 10.27 [9.97, 10.59]	1532 8.60 [8.26, 8.95]	968 16.10 [15.34, 16.90]	1017 27.67 [26.48, 28.90]	364 26.65 [24.73, 28.66]
Prescription of psychotropics	6188 11.88 [11.65, 12.12]	1082 41.55 [39.97, 43.15]	0 0.00 [0.00, 3.72]	58 0.23 [0.18, 0.28]	849 4.76 [4.51, 5.03]	2053 34.15 [33.16, 35.17]	3090 84.08 [83.06, 85.05]	1220 89.31 [87.86, 90.61]
Other events	35755 68.65 [68.32, 68.99]	1893 72.70 [71.24, 74.11]	66 94.29 [87.87, 97.41]	20141 78.24 [77.81, 78.66]	9699 54.43 [53.82, 55.05]	3324 55.30 [54.24, 56.35]	3205 87.21 [86.28, 88.09]	1213 88.80 [87.32, 90.13]
GP events	36763 70.59 [70.26, 70.92]	1965 75.46 [74.05, 76.82]	69 98.57 [93.85, 99.68]	20472 79.52 [79.10, 79.93]	9935 55.76 [55.15, 56.37]	3540 58.89 [57.84, 59.93]	3421 93.09 [92.37, 93.75]	1291 94.51 [93.40, 95.44]
Hospital admissions	3499 6.72 [6.54, 6.90]	493 18.93 [17.70, 20.23]	0 0.00 [0.00, 3.72]	515 2.00 [1.86, 2.15]	1659 9.31 [8.96, 9.68]	674 11.21 [10.56, 11.90]	659 17.93 [16.91, 19.00]	485 35.51 [33.41, 37.66]

Table A11: Incidence of the 1Y time-frame factors (individuals presenting each factor) for cases, controls and risk scores.

Factor	Controls	Cases	VLR	LR	MLR	MHR	HR	VHR
	N % [95% CI]	N % [95% CI]	N % [95% CI]	N % [95% CI]	N % [95% CI]	N % [95% CI]	N % [95% CI]	N % [95% CI]
**Total**	**52080**	**2604**	**70**	**25744**	**17818**	**6011**	**3675**	**1366**
Depression & anxiety	1185 2.28 [2.17, 2.39]	304 11.67 [10.68, 12.75]	0 0.00 [0.00, 3.72]	71 0.28 [0.23, 0.34]	247 1.39 [1.25, 1.54]	294 4.89 [4.45, 5.37]	457 12.44 [11.57, 13.36]	420 30.75 [28.73, 32.84]
Other common mental disorders	183 0.35 [0.31, 0.40]	43 1.65 [1.29, 2.12]	0 0.00 [0.00, 3.72]	62 0.24 [0.20, 0.30]	48 0.27 [0.21, 0.34]	35 0.58 [0.44, 0.77]	40 1.09 [0.84, 1.41]	41 3.00 [2.33, 3.86]
Other mental health	341 0.65 [0.60, 0.72]	45 1.73 [1.36, 2.20]	2 2.86 [0.95, 8.27]	134 0.52 [0.45, 0.60]	102 0.57 [0.49, 0.67]	46 0.77 [0.60, 0.97]	66 1.80 [1.47, 2.19]	36 2.64 [2.01, 3.45]
Unintentional injury&poisoning	2113 4.06 [3.92, 4.20]	181 6.95 [6.17, 7.82]	6 8.57 [4.50, 15.73]	993 3.86 [3.66, 4.06]	690 3.87 [3.64, 4.12]	224 3.73 [3.35, 4.15]	239 6.50 [5.87, 7.20]	142 10.40 [9.11, 11.83]
Self-harm	53 0.10 [0.08, 0.13]	131 5.03 [4.37, 5.78]	0 0.00 [0.00, 3.72]	2 0.01 [0.00, 0.02]	21 0.12 [0.08, 0.17]	17 0.28 [0.19, 0.42]	33 0.90 [0.68, 1.19]	111 8.13 [6.99, 9.43]
Alcohol misuse	171 0.33 [0.29, 0.37]	96 3.69 [3.13, 4.34]	0 0.00 [0.00, 3.72]	12 0.05 [0.03, 0.07]	42 0.24 [0.18, 0.30]	39 0.65 [0.50, 0.84]	52 1.41 [1.13, 1.77]	122 8.93 [7.74, 10.28]
Drugs misuse	135 0.26 [0.23, 0.30]	63 2.42 [1.97, 2.97]	0 0.00 [0.00, 3.72]	13 0.05 [0.03, 0.08]	29 0.16 [0.12, 0.22]	30 0.50 [0.37, 0.67]	38 1.03 [0.79, 1.35]	88 6.44 [5.43, 7.62]
Possible maltreatment	61 0.12 [0.09, 0.14]	18 0.69 [0.47, 1.01]	0 0.00 [0.00, 3.72]	24 0.09 [0.07, 0.13]	25 0.14 [0.10, 0.19]	10 0.17 [0.10, 0.28]	9 0.24 [0.14, 0.42]	11 0.81 [0.49, 1.31]
Non-physical sleep disorder	235 0.45 [0.41, 0.50]	36 1.38 [1.05, 1.81]	1 1.43 [0.32, 6.15]	58 0.23 [0.18, 0.28]	58 0.33 [0.26, 0.40]	57 0.95 [0.76, 1.18]	65 1.77 [1.44, 2.16]	32 2.34 [1.76, 3.12]
Physical sleep disorder	671 1.29 [1.21, 1.37]	32 1.23 [0.92, 1.64]	20 28.57 [20.62, 38.12]	438 1.70 [1.57, 1.84]	114 0.64 [0.55, 0.75]	67 1.11 [0.91, 1.36]	47 1.28 [1.01, 1.62]	17 1.24 [0.84, 1.84]
Prescription of opiates	6399 12.29 [12.05, 12.53]	449 17.24 [16.06, 18.49]	30 42.86 [33.57, 52.67]	2898 11.26 [10.94, 11.59]	1548 8.69 [8.35, 9.04]	1002 16.67 [15.89, 17.48]	1019 27.73 [26.53, 28.96]	351 25.70 [23.80, 27.69]
Prescription of psychotropics	6300 12.10 [11.86, 12.33]	937 35.98 [34.45, 37.54]	0 0.00 [0.00, 3.72]	374 1.45 [1.34, 1.58]	1113 6.25 [5.95, 6.55]	1909 31.76 [30.78, 32.75]	2782 75.70 [74.52, 76.85]	1059 77.53 [75.61, 79.33]
Other events	37211 71.45 [71.12, 71.77]	1866 71.66 [70.18, 73.09]	70 100.00 [96.28, 100.00]	22151 86.04 [85.68, 86.39]	9348 52.46 [51.85, 53.08]	3161 52.59 [51.53, 53.64]	3173 86.34 [85.38, 87.25]	1174 85.94 [84.33, 87.42]
GP events	38127 73.21 [72.89, 73.53]	1926 73.96 [72.52, 75.35]	70 100.00 [96.28, 100.00]	22518 87.47 [87.13, 87.80]	9557 53.64 [53.02, 54.25]	3308 55.03 [53.98, 56.09]	3349 91.13 [90.33, 91.87]	1251 91.58 [90.26, 92.74]
Hospital admissions	3895 7.48 [7.29, 7.67]	461 17.70 [16.51, 18.97]	2 2.86 [0.95, 8.27]	889 3.45 [3.27, 3.65]	1743 9.78 [9.42, 10.15]	632 10.51 [9.88, 11.18]	647 17.61 [16.60, 18.66]	443 32.43 [30.38, 34.55]

Table A12: Incidence of the 5Y time-frame factors (individuals presenting each factor) for cases, controls and risk scores.

**Factor**	**Controls** N % [95% CI]	**Cases** N % [95% CI]	**VLR** N % [95% CI]	**LR** N % [95% CI]	**MLR** N % [95% CI]	**MHR** N % [95% CI]	**HR** N % [95% CI]	**VHR** N % [95% CI]
**Total**	**52080**	**2604**	**70**	**25744**	**17818**	**6011**	**3675**	**1366**
Depression & anxiety	5645 10.84 [10.62, 11.07]	853 32.76 [31.26, 34.29]	0 0.00 [0.00, 3.72]	403 1.57 [1.44, 1.70]	2016 11.31 [10.93, 11.71]	1333 22.18 [21.31, 23.07]	1765 48.03 [46.67, 49.38]	981 71.82 [69.77, 73.77]
Other common mental disorders	1254 2.41 [2.30, 2.52]	180 6.91 [6.14, 7.78]	7 10.00 [5.51, 17.46]	436 1.69 [1.57, 1.83]	339 1.90 [1.74, 2.08]	205 3.41 [3.05, 3.82]	261 7.10 [6.44, 7.83]	186 13.62 [12.16, 15.21]
Other mental health	1800 3.46 [3.33, 3.59]	196 7.53 [6.72, 8.42]	0 0.00 [0.00, 3.72]	582 2.26 [2.11, 2.42]	650 3.65 [3.42, 3.89]	222 3.69 [3.31, 4.11]	341 9.28 [8.52, 10.10]	201 14.71 [13.21, 16.36]
Unintentional injury&poisoning	12793 24.56 [24.26, 24.88]	853 32.76 [31.26, 34.29]	14 20.00 [13.32, 28.91]	5310 20.63 [20.21, 21.04]	5086 28.54 [27.99, 29.10]	1292 21.49 [20.64, 22.38]	1269 34.53 [33.25, 35.83]	675 49.41 [47.19, 51.64]
Self-harm	441 0.85 [0.78, 0.92]	351 13.48 [12.42, 14.62]	0 0.00 [0.00, 3.72]	1 0.00 [0.00, 0.02]	39 0.22 [0.17, 0.28]	126 2.10 [1.81, 2.42]	208 5.66 [5.06, 6.32]	418 30.60 [28.59, 32.69]
Alcohol misuse	772 1.48 [1.40, 1.57]	272 10.45 [9.50, 11.47]	0 0.00 [0.00, 3.72]	9 0.03 [0.02, 0.06]	175 0.98 [0.87, 1.11]	251 4.18 [3.77, 4.62]	226 6.15 [5.53, 6.83]	383 28.04 [26.08, 30.08]
Drugs misuse	508 0.98 [0.91, 1.05]	205 7.87 [7.05, 8.78]	0 0.00 [0.00, 3.72]	13 0.05 [0.03, 0.08]	98 0.55 [0.47, 0.65]	145 2.41 [2.11, 2.76]	160 4.35 [3.83, 4.94]	297 21.74 [19.96, 23.63]
Possible maltreatment	505 0.97 [0.90, 1.04]	88 3.38 [2.84, 4.01]	0 0.00 [0.00, 3.72]	92 0.36 [0.30, 0.42]	235 1.32 [1.19, 1.47]	102 1.70 [1.44, 1.99]	91 2.48 [2.09, 2.93]	73 5.34 [4.43, 6.44]
Non-physical sleep disorder	1439 2.76 [2.65, 2.88]	175 6.72 [5.96, 7.57]	2 2.86 [0.95, 8.27]	408 1.58 [1.46, 1.72]	426 2.39 [2.21, 2.59]	255 4.24 [3.83, 4.69]	341 9.28 [8.52, 10.10]	182 13.32 [11.88, 14.91]
Physical sleep disorder	2070 3.97 [3.84, 4.12]	91 3.49 [2.95, 4.14]	51 72.86 [63.39, 80.63]	1346 5.23 [5.00, 5.46]	326 1.83 [1.67, 2.00]	211 3.51 [3.14, 3.92]	167 4.54 [4.01, 5.14]	60 4.39 [3.57, 5.40]
Prescription of opiates	15742 30.23 [29.90, 30.56]	901 34.60 [33.08, 36.15]	63 90.00 [82.54, 94.49]	8672 33.69 [33.20, 34.17]	3674 20.62 [20.13, 21.12]	1776 29.55 [28.59, 30.52]	1810 49.25 [47.90, 50.61]	648 47.44 [45.22, 49.66]
Prescription of psychotropics	11375 21.84 [21.55, 22.14]	1247 47.89 [46.28, 49.50]	0 0.00 [0.00, 3.72]	1716 6.67 [6.41, 6.93]	4007 22.49 [21.98, 23.01]	2635 43.84 [42.79, 44.89]	3064 83.37 [82.34, 84.36]	1200 87.85 [86.32, 89.23]
Other events	49544 95.13 [94.97, 95.28]	2387 91.67 [90.73, 92.51]	70 100.00 [96.28, 100.00]	25702 99.84 [99.79, 99.87]	17342 97.33 [97.12, 97.52]	3885 64.63 [63.61, 65.64]	3602 98.01 [97.60, 98.36]	1330 97.36 [96.55, 97.99]
GP events	49436 94.92 [94.76, 95.08]	2286 87.79 [86.69, 88.80]	70 100.00 [96.28, 100.00]	25744 100.00 [99.99, 100.00]	17434 97.84 [97.66, 98.02]	3672 61.09 [60.05, 62.12]	3499 95.21 [94.60, 95.76]	1303 95.39 [94.36, 96.24]
Hospital admissions	16988 32.62 [32.28, 32.96]	1297 49.81 [48.20, 51.42]	6 8.57 [4.50, 15.73]	5665 22.01 [21.58, 22.43]	7178 40.29 [39.68, 40.89]	2208 36.73 [35.72, 37.76]	2205 60.00 [58.66, 61.32]	1023 74.89 [72.91, 76.77]
